# Editorial: Appetite Control in Obesity

**DOI:** 10.3389/fnut.2022.959627

**Published:** 2022-06-28

**Authors:** Alessio Molfino, Giovanni Imbimbo

**Affiliations:** Department of Translational and Precision Medicine, Sapienza University of Rome, Rome, Italy

**Keywords:** obesity, overweight, appetite, energy homeostasis, therapy

Obesity represents a worldwide major health problem with a prevalence growing exponentially in the last decades (1). This phenomenon increases the major risk factors for developing several chronic conditions such as diabetes, cardiovascular disease, cancer and chronic kidney disease (1, 2). Importantly, obesity and overweight in the adult population have been associated with a strong reduction in life expectancy and with increased early mortality (3). In adulthood, obesity was found to be a powerful predictor of mortality also at older ages (4). In parallel, obesity determines an important economic burden on nations and on single individuals (5) with a global economic impact that was estimated in 2030 to be around US $2.0 trillion or ~3% of the global gross domestic product (6, 7).

From a pathophysiological point of view, the obesity phenotype is the consequence of a long-term altered energy balance with an increased energy intake and decreased calorie expenditure (8, 9).

The mechanisms are multifactorial, including interaction among genetic, epigenetic, physiological and psychological aspects with the environment (8). A crucial factor is represented by the modulation of energy homeostasis mediated by the central nervous system (8), and appetite is mainly regulated by three systems: (i) the Agouti-related protein (AGRP) neurons that are located in the hypothalamic arcuate nucleus (ARC) and stimulating food intake; (ii) neurons in lateral hypothalamus that are involved in positive feedback for increasing food assumption; (iii) neurons in the parabrachial nucleus that are potent suppressors of food intake. This system physiologically interacts with internal and external stimuli regulating the energy balance (8, 10, 11).

In this light, the understanding of the mechanisms underlying appetite regulation/dysregulation is of great interest in order to develop new therapeutic strategies to counteract obesity and the associated negative consequences.

Altered appetite represents the substrate for different diseases that may lead to two opposite phenotypes, i.e., obesity and malnutrition. Except for rare genetic conditions, such as MC4R deficiency, hyperphagia in obese patients is often driven mainly by different environmental factors, including dietary patterns (in particular, western diets), abundance of high palatable food and to psychological aspects (e.g., anxiety, lack of sleep) influencing the daily calorie intakes (9). However, excluding the rare monogenic syndromes, in obesity genome wide association studies (GWAS) identified different loci associated with an obese phenotype which may represent a genetic predisposition (12, 13). In particular, a recent study found 112 new loci that were not previously identified among the ones associated with high body mass index (BMI) in the Japanese population (12). Interestingly, this study showed a genetic correlation between lymphocyte count and BMI, providing insights in body weight regulation and lymphocytes to be furtherly investigated (12).

By GWAS studies, glucosamine-6-phosphate deaminase (GNPDA2) was associated with obesity (14) and in a recent article appeared in the present Research Topic of *Frontiers in Nutrition*, Gutierrez-Aguilar et al. observed in an animal model that, although GNPDA2 seemed not involved in appetite regulation at central level, it may play a role in glucose homeostasis.

Also, the fat mass and obesity-related (FTO) variants were extensively investigated due to their association(s) with increased BMI. In fact, the FTO gene variants were considered important determinants of impaired energy balance and, in turn, obesity (15).

More recently, several studies focused on the epigenetic mechanisms as contributor for the development of different metabolic derangements including obesity (16). Interestingly, the evaluation of epigenetic alterations in BDNF promoter in children showed a preliminary association with appetite modulation (17). In this light, the study of the epigenetics mechanisms regarding appetite regulation may explain the interaction between environment and body weight homeostasis. Importantly, the understanding of human epigenome and its interaction with the pathogenesis of obesity may provide advanced and personalized therapeutic strategies.

Interestingly, different molecules regulating energy homeostasis were described to be able to modulate appetite in different settings. For instance, the growth differentiation factor 15 (GDF15)—an inflammatory cytokine—was initially investigated for its role in pathophysiology of cancer anorexia (18, 19). In particular, a recent study showed that GDF15 determined reduced appetite and, in turn low food intake binding in the area postrema and nucleus of the solitary tract the GDNF family receptor α-like (GFRAL) and its co-receptor Ret proto-oncogene (RET) (20). In this light, the GDF15-GFRAL axis was considered for obesity therapy showing promising results in animal studies (21). Moreover, GDF15 may have anti-obese effects determining fat loss inducing thermogenesis (22).

Also, the Lipocalin-2 (LCN2) is a novel bone-derived mediator able to suppress food intake by the interaction with the melanocortin receptor 4 (MC4R) in the hypothalamus (23). The role of LCN2 was also investigated in the setting of obesity (24) showing that LCN2 may act by the modulation of appetite and by inducing β-cell proliferation, serving as compensatory signal (25). Elucidating the role of bone in appetite control in obesity may clarify the role of LCN2 in this setting.

Interestingly, Maric et al. analyzed using experimental models (mice and rats) potential differences of diet-induced obesity according to sex and found that male and female animals responded in a different way to high fat diet by different metabolic compensatory pathways and this divergence was seen also between mice and rats.

In addition, the role of some vitamins (vitamin E and K2) have been investigated in metabolic syndrome and in fat metabolism by Zhang et al. and Qu et al.

Nowadays, different drugs were approved by FDA for obesity treatments (24), including phentermine, lorcaserin, naltrexone and liraglutide acting on CNS reducing appetite, and the orlistat decreasing fat absorption (24). Although these drugs showed capability to reduce body weight in clinical trials, the efficacy of these therapies is still considered limited. On the other side, several drugs, in particular antipsychotics, may dramatically affect appetite and understating their effects on energy homeostasis have been well analyzed by Mukherjee et al., focusing on the receptor binding profile of these drugs.

For all these considerations, the interest of appetite regulation in obesity analyzed in the present Research Topic may highlight new pathways involved in obesity and their role for the development of novel personalized anti-obesity treatments.

## Author Contributions

AM and GI wrote the article. Both authors contributed to manuscript revision, read, and approved the submitted version.

## Conflict of Interest

The authors declare that the research was conducted in the absence of any commercial or financial relationships that could be construed as a potential conflict of interest.

## Publisher's Note

All claims expressed in this article are solely those of the authors and do not necessarily represent those of their affiliated organizations, or those of the publisher, the editors and the reviewers. Any product that may be evaluated in this article, or claim that may be made by its manufacturer, is not guaranteed or endorsed by the publisher.
